# A novel human donor cornea preservation cocktail incorporating a thermo-reversible gelation polymer (TGP), enhancing the corneal endothelial cell density maintenance and explant culture of corneal limbal cells

**DOI:** 10.1007/s10529-021-03116-y

**Published:** 2021-03-25

**Authors:** Bhuvaneshwari Namitha, Munusamy Rajendran Chitra, Mathevan Bhavya, Periasamy Parikumar, Shojiro Katoh, Hiroshi Yoshioka, Masaru Iwasaki, Rajappa Senthilkumar, Mathaiyan Rajmohan, Ramalingam Karthick, Senthilkumar Preethy, Samuel J. K. Abraham

**Affiliations:** 1 Regional Institute of Ophthalmology, Egmore, Chennai, Tamilnadu India; 2 The Light Eye Hospital, Salem Main Rd, Dharmapuri, Tamil Nadu 636701 India; 3 Edogawa Evolutionary Lab of Science (EELS), Edogawa Hospital Campus, 2-24-18, Higashi Koiwa, Edogawa-Ku, Tokyo 133-0052 Japan; 4 Mebiol Inc., 1-25-8, Nakahara, Hiratsuka, Kanagawa 254-0075 Japan; 5 Centre for Advancing Clinical Research (CACR), Faculty of Medicine, Yamanashi University, 1110, Shimokato, Chuo, Yamanashi 409-3898 Japan; 6 The Fujio-Eiji Academic Terrain (FEAT), Nichi-In Centre for Regenerative Medicine (NCRM), PB 1262, Chennai, Tamil Nadu 600034 India; 7 The Mary-Yoshio Translational Hexagon (MYTH), Nichi-In Centre for Regenerative Medicine (NCRM), PB 1262, Chennai, Tamil Nadu 600034 India; 8 JBM Inc., 3-1-14, Higashi Koiwa, Edogawa-Ku, Tokyo 133-0052 Japan; 9 GN Corporation Co. Ltd., 3-8, Wakamatsu, Kofu, Yamanashi 400-0866 Japan

**Keywords:** Corneal storage, MK medium, Optisol—GS, TGP, Corneal limbal cell culture

## Abstract

**Purpose:**

McCarey-Kaufman’s (MK) medium and Optisol-GS medium are the most commonly employed media for human donor corneal preservation. In this study, we evaluated the preservation efficacy of discarded human donor corneas using a Thermo-reversible gelation polymer (TGP) added to these two media.

**Methods:**

Thirteen human corneal buttons collected from deceased donors, which were otherwise discarded due to low endothelial cell density (ECD) were used. They were stored in four groups: MK medium, MK medium with TGP, Optisol-GS and Optisol-GS with TGP at 4 °C for 96 h. Slit lamp examination and specular microscopy were performed. Corneal limbal tissues from these corneas were then cultured using explant methodology one with and the other without TGP scaffold, for 21 days.

**Results:**

MK + TGP and Optisol-GS + TGP preserved corneas better than without TGP, which was observed by maintenance of ECD which was significantly higher in Optisol-GS + TGP than MK + TGP (p-value = 0.000478) and corneal thickness remaining the same for 96 h. Viable corneal epithelial cells could be grown from the corneas stored only in MK + TGP and Optisol-GS + TGP. During culture, the TGP scaffold helped maintain the native epithelial phenotype and progenitor/stem cell growth was confirmed by RT-PCR characterization.

**Conclusion:**

TGP reconstituted with MK and Optisol—GS media yields better preservation of human corneal buttons in terms of relatively higher ECD maintenance and better in vitro culture outcome of corneal limbal tissue. This method has the potential to become a standard donor corneal transportation-preservation methodology and it can also be extended to other tissue or organ transportation upon further validation.

## Introduction

Corneal blindness is a major global ophthalmic public health problem with a prevalence of 4.9 million cases (Oliva et al. 2012). In India, with an estimated 1.2 million corneally blind persons at any point of time, 25 000–30 000 people are added to this list every year (National Programme for Control of Blindness). Corneal transplantation remains the definitive treatment for the condition. Meanwhile, it is estimated that 270 000 donor eyes are required to perform 100 000 corneal transplants per year in India, which is 20 times higher than the actual number of corneas available from donors in India (Saini 1997). Among 20,564 donor eyes collected by 12 eye banks during the years 2013–2014 in India, only 50.5% could be used for transplantation. Of this 50.5%, 40.36% were non-optical grade, whereas 59.64% were optical grade (Sharma et al. 2019). Developed countries like New Zealand report a high utilization rate of 88% of donor corneas collected (Sharma et al. 2019). A study from India in 2019 reported that among various causes that affect donated corneal utilization, the storage medium used played a major role. While the most commonly used storage medium in India is MK media (83.33%), media like Optisol—GS are most commonly used in developed countries which have better corneal preservation capacities (Sharma et al. 2019). MK medium is capable of storing the cornea for 3–4 days (McCarey and Kaufman 1974); Optisol—GS medium is capable of storing the cornea for 14 days with better endothelial cell density (Kaufman et al. 1991), which is considered as the major criterion to assess the suitability of the cornea for utilization (Jan and Peter 2009). We have earlier reported the usage of a thermo-reversible gelation polymer (TGP) reconstituted in DMEM (Dulbecco’s modified Eagle’s medium) for the transportation of corneal endothelial tissue whose cell viability could be maintained for up to 72 h of storage without cool preservation to be used for in vitro corneal endothelial progenitor cell expansion and transplantation to treat corneal endothelial diseases (Rao et al. 2014; Parikumar et al. 2014, 2018). Herein we report the suitability of TGP in preserving donor corneas with superior characteristics when reconstituted with MK medium and Optisol—GS medium.

## Materials and methods

Thirteen discarded corneal button samples collected from deceased donors aged between 29 and 93 years, obtained from the Regional Institute of Ophthalmology in Egmore, Chennai, India after institutional ethics committee approval were used in the study. These corneal buttons were employed because they were discarded as unfit for transplant either due to infection or due to endothelial cell density (ECD) less than the prescribed standard.

For the study, the corneal buttons were subjected to slit lamp examination and specular microscopic examination once again.

McCarey and Kaufman (MK) medium was provided by the Government Ophthalmic Hospital, Egmore, Chennai, India. Optisol—GS was purchased from Bausch & Lomb Surgical, Irvine, CA, USA and TGP from GN Corporation, Japan through Nichi-In Biosciences Pvt. Ltd., India.

### Reconstitution of TGP

Lyophilized TGP vials (1 g) were provided by Nichi-In Biosciences Pvt. Ltd (manufactured by Mebiol Inc., Japan; procured by M/s GN Corporation, Japan). One gram of TGP was reconstituted with 10 ml in MK medium and another TGP was reconstituted with 10 ml in Optisol-GS and incubated at 4 °C overnight. This reconstitution was performed one day prior to sample preservation.

### Experimental groups

The study comprised four groups of preservation media: MK medium, TGP reconstituted with MK medium, Optisol-GS and TGP reconstituted with Optisol-GS. The thirteen corneal buttons were split for preservation in the following groups.

Group I: Four (n = 4) corneal buttons were immersed in 12 ml of MK medium (MK).
Group II: Three (n = 3) corneal buttons were embedded in 4 ml of cooled TGP reconstituted with MK medium and allowed to solidify at room temperature for five minutes. Eight millilitres of MK medium were then added over the TGP (MK + TGP).
Group III: Three (n = 3) corneal buttons were immersed in 12 ml of Optisol-GS medium (Optisol—GS).
Group IV: Three (n = 3) corneal buttons were embedded in 4 ml of cooled TGP reconstituted with Optisol-GS and allowed to solidify at room temperature for five minutes. 8 ml of Optisol—GS medium was added over the solidified TGP. (Optisol—GS + TGP).

The corneal buttons were preserved in their respective groups in polystyrene boxes for 96 h at 4 °C. After 96 h’ preservation, each corneal button was subjected to slit lamp examination and specular microscopic examination.

### Slit-lamp examination & specular microscopic examination

Each cornea was evaluated using slit-lamp examination and specular microscopic examination (Kerato Analyzer EKA-10, KONAN©) pre- and post-preservation. Slit-lamp examination comprised an assessment of all layers of the cornea, with the epithelium being assessed for scars, oedema and arcus (Saini et al. 1996). Specular microscopic examination was performed, with cell density recorded as the number of cells per square millimetre.

### Limbal explant culture

After the preservation, each corneal button was subjected to limbal epithelial stem cell culture (Sudha et al. 2006; Sitalakshmi et al. 2009). Briefly, the corneal button was placed inside a sterile tissue culture dish, and four to five limbal tissue bits, each measuring 2 × 2 mm^2^, were cut from the tissue. The tissue bits were transferred to fresh tissue culture dishes for further mincing into small pieces immersed in a few drops of phosphate buffer solution (PBS; Gibco-Thermo, Fisher Scientific, Waltham, MA, USA) to avoid tissue drying. The tissue bits were divided into two halves; one portion was seeded in a TGP-based culture methodology, termed as three-dimensional (3D)-TGP), while the other was cultured without the TGP scaffold (Sudha et al. 2006; Sitalakshmi et al. 2009), termed as two-dimensional (2D) culture. The culture medium used in both groups consisted of Dulbecco’s modified Eagle’s medium (DMEM; Gibco-Thermo, Fisher Scientific, Waltham, MA, USA) with 10% foetal bovine serum (FBS).

For the TGP culture, TGP was reconstituted in DMEM. A drop of TGP-reconstituted DMEM medium (TGP–DMEM) was placed in the centre of a 12-well tissue culture plate (Corning Inc., Corning, NY) and solidified at 37 °C. Each tissue bit was carefully placed in the well plate, following which a drop of TGP–DMEM was placed to cover the tissue bit. The tissue bits were sandwiched by TGP-DMEM with further addition of 200 μL of the culture medium. All tissue culture plates were placed in a humidified CO_2_ incubator at 37 °C. The cells were cultured for 21 days, and media change was done twice per week.

Total RNA was isolated from the explant cultures in TGP scaffold alone, as viable cells could not be retrieved from the conventional 2D cultures. Cells were collected, treated with TriReagent (Sigma Aldrich, St Louis, USA) according to the manufacturer’s recommended protocol and total RNA was extracted and stored at − 80 °C until use (Sudha et al. 2006). Reverse transcription was performed using sensiscript reverse transcripatase (Qiagen, Germany). PCR amplifications of the first-strand cDNAs were performed using specific primer pairs for housekeeping gene, glyceraldehyde-3 phosphate dehydrogenase (GAPDH) as internal control, and markers DNp63 and Connexin 43 in the Eppendorf PCR systems. PCR products were fractionated by electrophoresis using 2% agarose gel containing 0.5% ethidium bromide with molecular marker Hin*f* I φ Fdigest to confirm the size of the resultant product (Sudha et al. 2006).

### Primer sequences employed (Sudha et al. 2006)

GAPDH

- Forward Primer: GCCAAGGTCATCCATGACAAC
- Reverse Primer: GTCCACCACCCTGTTGCTGTA

DNp63

- Forward Primer: CAGACTCAATTTAGTGAG
- Reverse Primer: AGCTCATGGTTGGGGCAC

Connexin 43

- Forward Primer: CCTTCTTGCTGATCCAGTGGTAC
- Reverse Primer: ACCAAGGACACCACCAGCAT

All data were analyzed using Excel software statistics package analysis software (Microsoft Office Excel®); Student’s paired *t*-tests were also calculated using this package; p-values < 0.05 were considered significant.

**Fig. 1:**
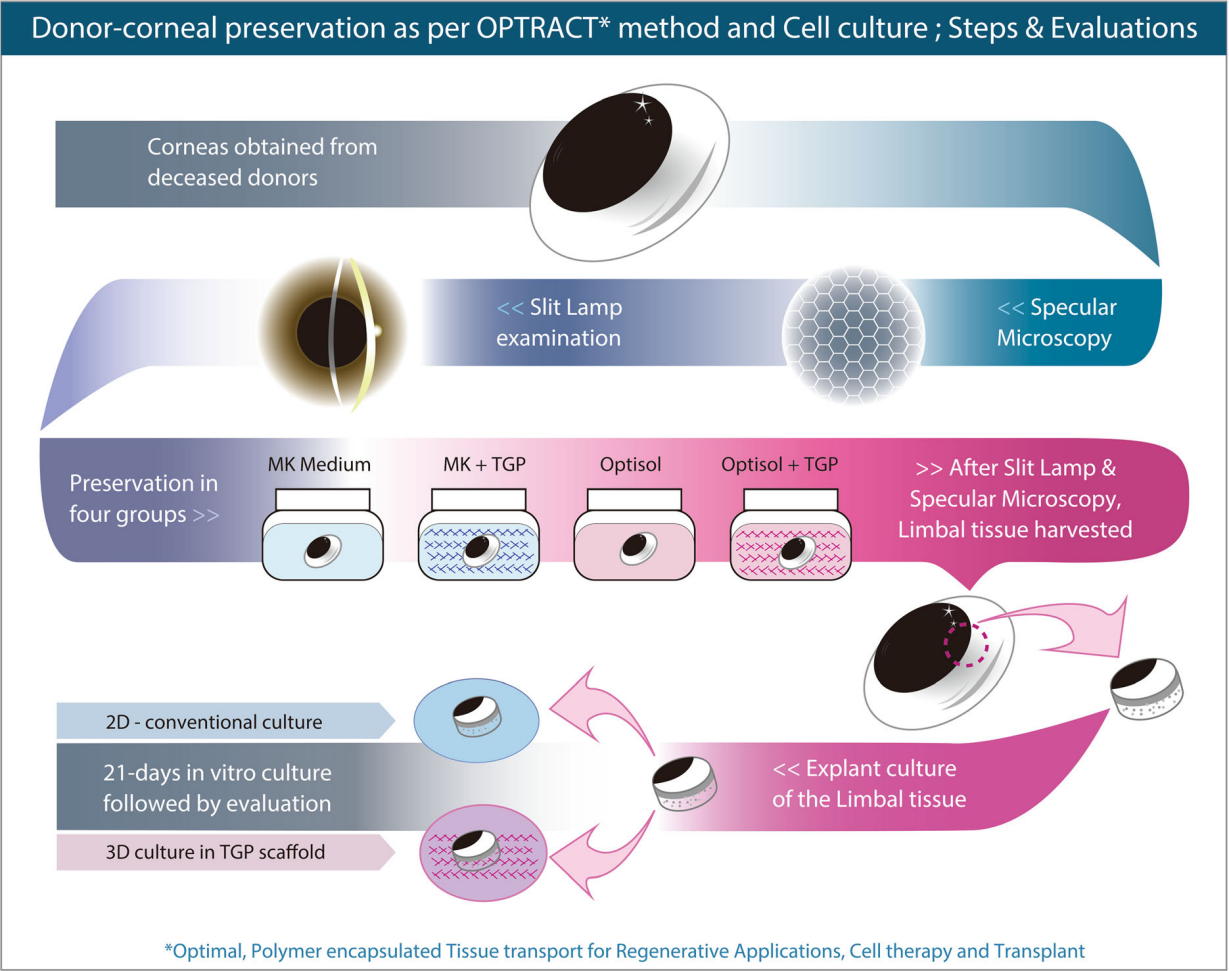
Schematic illustration of the outline of the various steps and evaluations performed in the study as per the OPTRACT (Optimal, Polymer encapsulated Tissue transport for Regenerative Applications, Cell therapy and Transplant) method

Figure 1 shows the schematic illustration of the various steps and evaluations performed in the study.

## Results

**Fig. 2:**
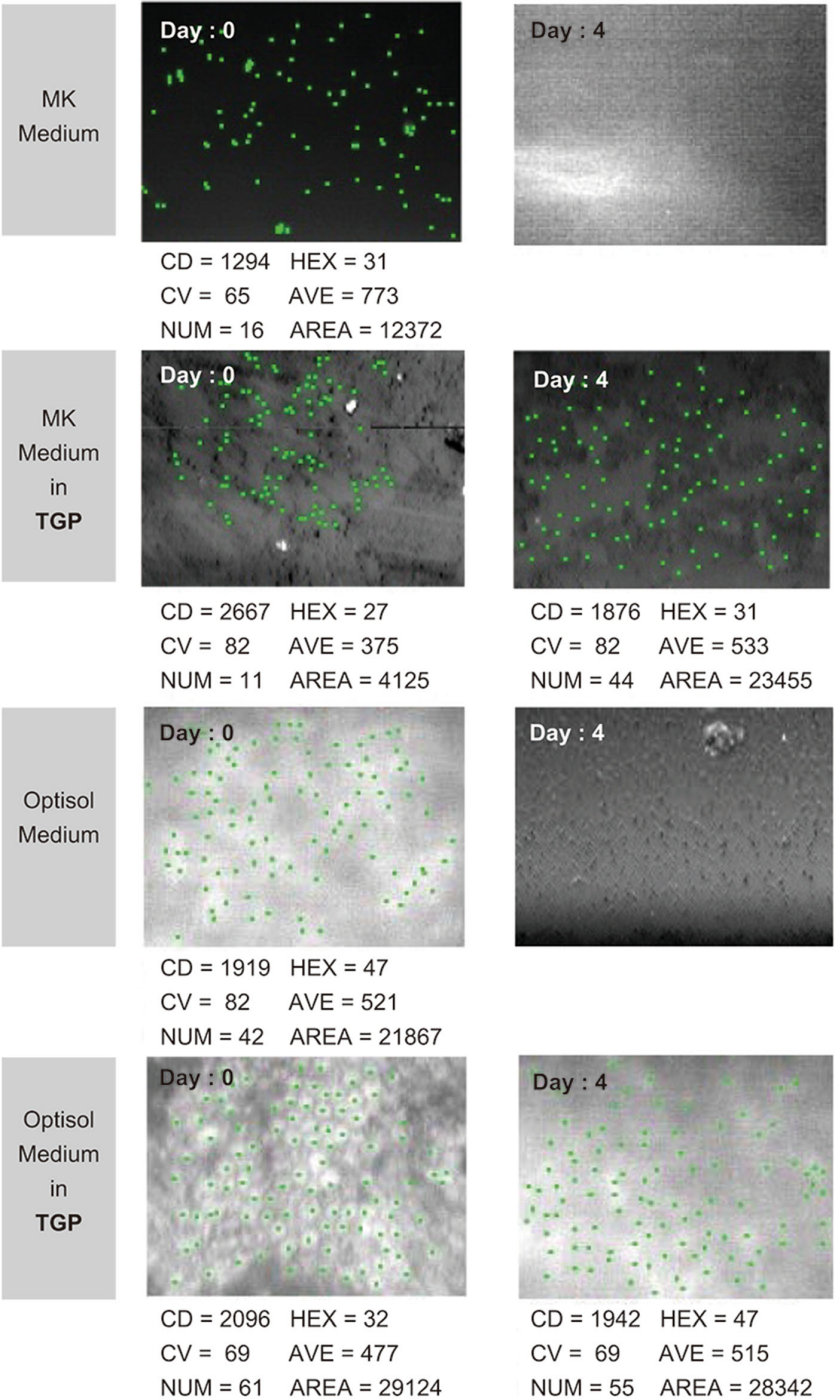
96 h pre- and post-preservation specular microscopy showing maintenance of endothelial cell density (ECD) only in the MK + TGP and Optisol-GS + TGP groups

The ages of the corneal button donors ranged from 29 to 92 years. The mean age was 56.1 years and median was 62 years (7 male and 6 female donors). There was no correlation between ECD maintenance and age, gender of the donor. Slit lamp examination of the corneas preserved for 4 days (96 h) revealed increases in stromal oedema and thickening in the corneas stored in the MK medium group. Stromal oedema, Descemet folds and thickening were the same before and after preservation in the MK + TGP, Optisol—GS and Optisol—GS + TGP groups. The endothelial cells were present only in the MK + TGP and Optisol—GS + TGP groups after preservation (Fig. 2). The ECD was an average of 2048 before storage in Optisol—GS + TGP while it was 1921 after storage. In MK + TGP group the ECD’s average before storage was 3682.5 and after storage it was 1775.5. The maintenance of ECD after storage in Optisol-GS + TGP was significantly higher than that of MK + TGP (p value = 0.000478) In the MK and Optisol—GS-only groups preserved for 96 h, the endothelial cells were either absent or too few to enumerate using specular microscopy.

**Fig. 3:**
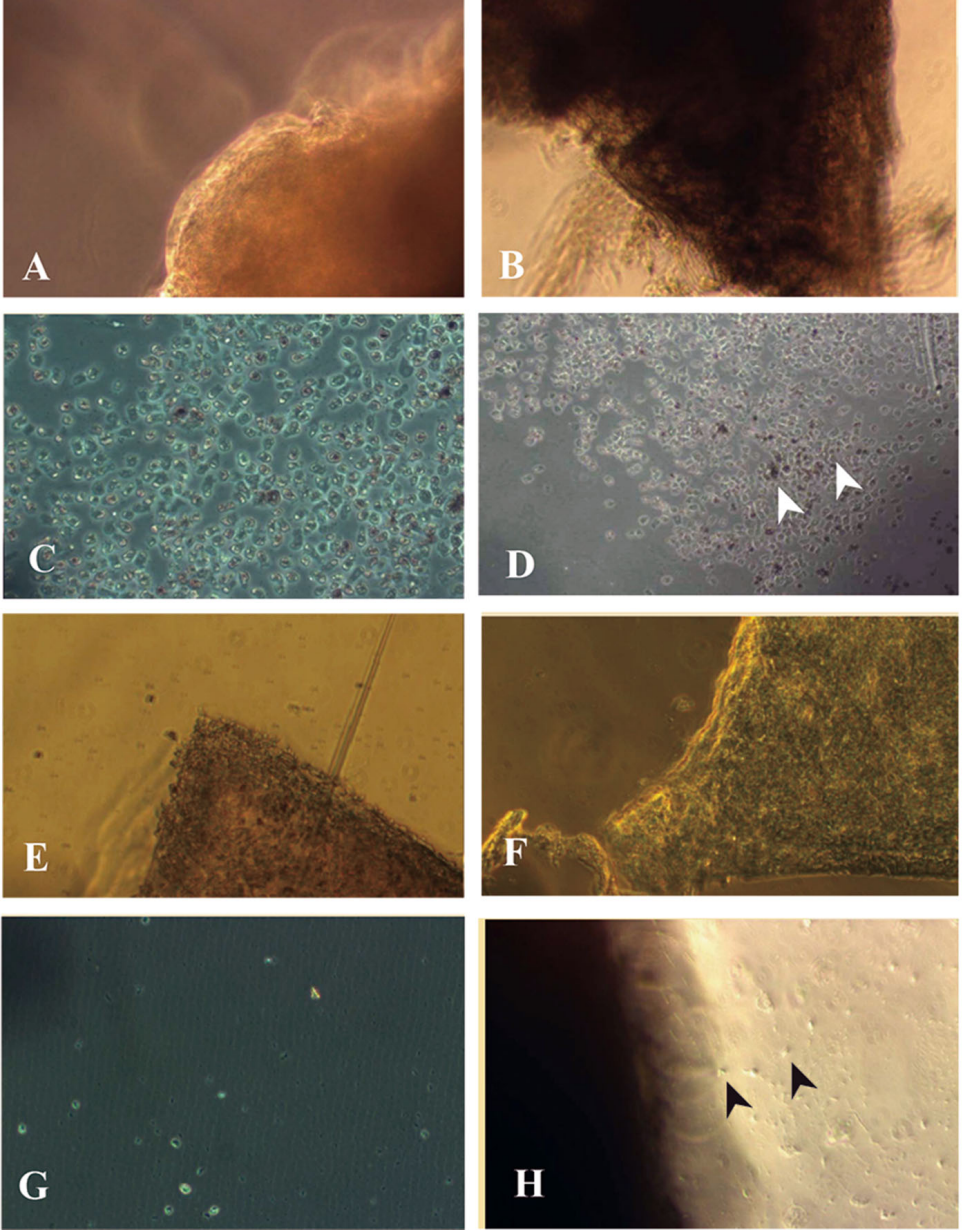
Explant cultures **A** and**B** MK Medium preserved;**A** Conventional culture; **B** TGP culture; **C** and **D** MK + TGP medium preserved; **C** Conventional culture showing cells grown; **D** Arrows in TGP culture showing cells grown as clusters; **E** and **F** Optisol- GS medium preserved; **E** Conventional culture; **F** TGP culture; **G** and **H** Optisol- GS + TGP preserved; **G** Conventional culture showing few cells grown; **H** Arrows in TGP culture showing rounded cells grown; Magnification: X 10

**Fig. 4:**
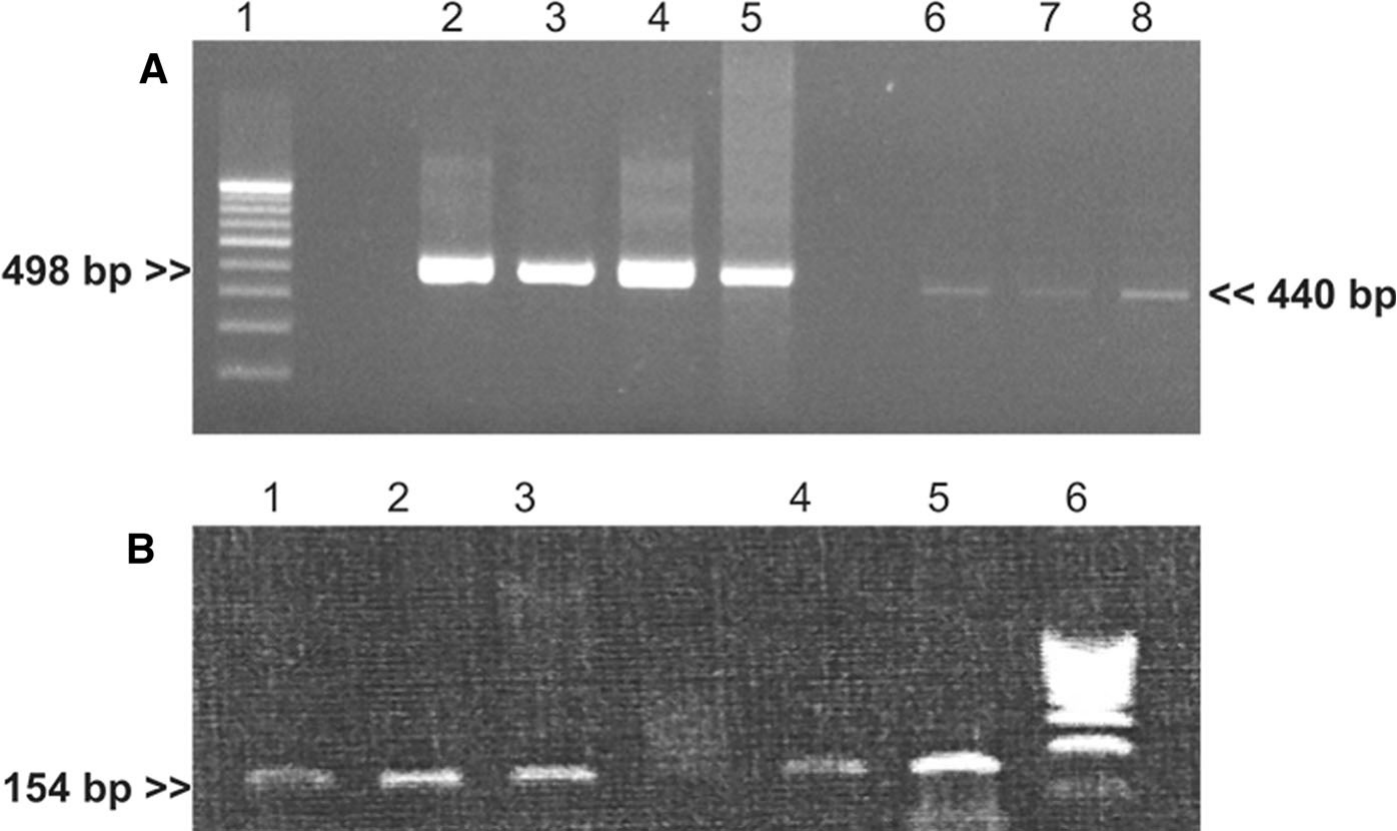
Results of reverse transcription-polymerase chain reaction (RT-PCR) for m-RNA expression of DNp63 and Connexin 43 on the cells grown in the TGP scaffold; **A** Lane 1: Molecular weight marker φ X Hinf I Digest; Lanes 2–5: GAPDH; Lanes 6–8—p63 positive cells grown in TGP; **B** Lanes 1–3—Connexin 43 positive TGP-grown cells; Lanes 4 & 5: Positive control; Lane 6 – Molecular weight marker φ X Hinf I Digest

In the explant culture, viable cells could be observed only in corneas preserved in the groups MK + TGP and Optisol—GS + TGP (Fig. 3). Especially in the cultures grown in 3D-TGP scaffold of tissues preserved in MK + TGP, the cells migrated out from the tissue, spreading to form a confluent culture with better maintenance of the native epithelial phenotype with several cell clusters (Fig. 3D). In the 2D culture, these clusters could not be observed. In the 3D-TGP culture of tissues preserved in Optisol—GS + TGP as well, cells were rounded and migrated out of tissue in the TGP scaffold-based culture, while only very minimal cells could be observed in the conventional 2D culture (Fig. 3H). In RT-PCR, Connexin 43 and p63 were positive only for the cells cultured in the TGP scaffold, proving that 3D-TGP helps in the growth of both transient amplifying cells (TACs; Connexin 43 positivity) and limbal stem cells (p63 positivity; see Fig. 4).

## Discussion

Preservation of human corneas for use in keratoplasty started in 1905, when the first cornea was kept in warm saline solution and immediately transplanted (Armitage et al. 2006; Zirm 1906). Storage of whole eyes in glass jars (equivalent to moist chambers) started in the 1930s (Armitage et al. 2006). With the world’s first eye bank established in the US in the 1930s, several techniques were pioneered to preserve corneas (Filatov 1935). The corneas were tested for the presence of infection and subjected to slit lamp examination. Further, ECD ranging from 2100 to 2500 cells/mm^2^ is considered as the widely applied method to assess the quality of corneas for transplantation (Armitage 2011). Hypothermic storage using preservation medium is extensively used in US eye banks and in developing countries such as India. Organ culture is widely employed in European eye banks in which the corneas are stored in Eagle’s minimum essential medium (MEM) with 2% foetal bovine serum (FBS) up to 8%. FBS is used by some eye banks with antibiotics for 1–2 weeks without change of the media (Basu 1995). In the case of corneal preservation media, several media, most containing chondroitin sulphate, have been developed. MK medium, which was first described by McCarey and Kaufman, is predominantly a mixture of tissue culture medium (T-C 199) and dextran (5%, 40,000 molecular weight). MK medium is widely used in developing countries such as India owing to its low-cost factor (Sharma et al. 2019; Basu 1995). The addition of chondroitin sulphate has helped increase storage time, leading to development of media like K-Sol (Cilco, Huntington, West Virginia), chondroitin sulphate storage medium (CSM), Dexsol, Optisol—GS (Chiron Ophthalmics Inc. Irvine, California) etc. Among these, Optisol—GS has been shown to preserve the corneal endothelium best up to 14 days, yielding a thinner cornea which permits better evaluation and tissue manipulation during surgery and faster visual rehabilitation (Basu 1995).

In our study, the addition of TGP has contributed to better preservation of the cornea by not allowing the thickness of the cornea to increase after storage and by maintaining the ECD. It is of note that the corneas used in the study were discarded due to lack of proper ECD or due to infection. Expansion of corneal epithelial cells was possible only in the corneas which were preserved by adding TGP to MK medium and Optisol—GS medium (Fig. 3). TGP has been shown to aid the transportation of corneal endothelial tissue (Rao et al. 2014) and retinal pigment epithelial tissue (Senthilkumar et al. 2012) without even cool preservation up to 72 h, in studies done by our team earlier. In those studies, cool preservation was not employed because maintenance of optimal temperature during cold-chain storage is still a problem in developing nations like India (Samant et al. 2007) where the study was performed. Though TGP was used with DMEM culture medium in that study and was not cool-preserved during transportation, in the current study we employed the conventional media already standardized for corneal preservation like the MK medium and Optisol-GS to which TGP was added to check if TGP can enhance the preservation properties of those media. Also, the preservation-transportation was done at 4 °C because the stability of those media (MK medium and Optisol-GS) at varying temperature has not been still explored. The current study, we performed as only a pilot experimentation and in future studies, we plan to study the preservation of the corneas with conventional corneal preservation media such as MK medium and Optisol-GS apart from cell culture medium with TGP at varying temperature instead of cool-preservation.

Cells obtained after transport in TGP have been expanded and transplanted to humans in pilot studies (Parikumar et al. 2018). TGP is available in lyophilized form and can be reconstituted with any culture medium or even fluids like saline. TGP helps to preserve native cell phenotypical characteristics better and also helps stem and progenitor cells to proliferate. These effects have been proven with both corneal epithelial and endothelial cells in previous studies  (Sitalakshmi et al. 2009; Sudha et al. 2006; Rao et al. 2014). In the current study as well, TGP scaffold-seeded cells helped preserve epithelial morphology better in culture. The cells grew as clusters (Fig. 2D) and with rounded shape (Fig. 2H) in TGP indicating an un-differentiated nature, presumed to be progenitor or stem cells which was proven by RT-PCR for Connexin 43 (TAC) and p63 for limbal stem cells (Sudha et al. 2006). Our study also suggests that the ability to retrieve viable cells by culturing post-preservation can be employed as a method for evaluating corneas’ suitability for clinical use. Limitations of the study include a limited number of samples and a lack of advanced evaluation techniques. We plan to overcome these in future studies by including characterization by biomarkers and electron microscopy examination of both the preserved corneas and the cells cultured from them, apart from studying for correlations between ECD with age and gender of donor and the implications of TGP-preservation related to the same. Further, pH of the medium which is a critical physiological parameter for effective maintenance of mammalian cells (Michl et al. 2019; Kisaka et al. 2015) was not measured before and after storage. Optisol has a baseline pH of 7.4 while it ranges from 6.46 to 7.33 after storage of corneal buttons for a period of 7–14 days (Sundaresan et al. 2019) MK medium has a baseline pH of 7.5 while it is around 8.2 after storage of corneal buttons for a period of 4 days (Hasany and Basu 1987). pH of TGP is neutral. TGP has been reconstituted with several media and the pH did not change beyond physiologically permissible limits for maintenance of the cells with any of the biological solutions used for the reconstitution (Madhavan et al. 2004; Kataoka and Huh 2010). Hence, we didn’t measure the pH of the storage medium when TGP was combined with MK medium or Optisol-GS but we do intend to perform this evaluation in future studies.

TGP enables in in vitro, a 3D environment for rapid cell growth, prevention of large cell aggregate formation, providing isolation of cells from shear forces, and sufficient porosity for nutrient diffusion (Lei and Schaffer 2013) (as oxygen and nutrient diffusion are critical parameters for maintenance of cell viability (Kisaka et al. 2017)), enabling cells to grow with their native phenotype maintained for a long period of time for different types of cells including pluripotent stem cells (Lei and Schaffer 2013), very sensitive corneal endothelial progenitors (Rao et al. 2014), avascular cartilage obtained chondrocytes (Arumugam et al. 2011; Katoh et al. 2020) etc. Since reconstitutions of TGP with different media are possible (Senthilkumar et al. 2012; Kataoka and Huh 2010; Nagaya et al. 2006; Lei and Schaffer 2013) and this study along with Rao et al. 2014 has shown that even the most sensitive tissues like the cornea can be preserved better by employing TGP, it will be worthwhile to consider trying TGP for storage or preservation and transportation of different tissues and organs for transplantation. We have termed this methodology, OPTRACT- Optimal, Polymer encapsulated Tissue transport for Regenerative Applications, Cell therapy and Transplant.

## Conclusion

When reconstituted using MK and Optisol—GS media, thermo-reversible gelation polymer (TGP) scaffold helps preserve donor corneal buttons with better ECD and corneal limbal tissue. Such preserved corneas yield viable cells in in vitro culture. Further validation of TGP-MK medium and TGP-Optisol—GS cocktails in larger studies are likely to yield a TGP-based cocktail for routine usage in preservation and transportation of donor corneal buttons for better outcomes of corneal transplantation procedures. When such TGP-based cocktail-transported corneal buttons yield better outcomes in the clinical setting, this preservation method may also be considered for transportation of other tissues and organs.
